# Supplementary material for: Observation of strong excitonic magneto-chiral anisotropy in twisted bilayer van der Waals crystals
Source: Nat Commun. 2021 Apr 7;12:2088. doi: 10.1038/s41467-021-22412-9 (PMC8027870; doi:10.1038/s41467-021-22412-9)
Supplement: Supplementary file 1 — Supplementary Information [file 41467_2021_22412_MOESM1_ESM.pdf]

Supplementary Information for

Observation of strong excitonic magneto-chiral anisotropy in twisted bilayer van der Waals crystals

Shoufeng Lan[1,2], Xiaoze Liu[1], Siqi Wang[1], Hanyu Zhu[1], Yawen Liu[3], Cheng Gong[1], Sui Yang[1], Jing Shi[3], Yuan Wang[1], Xiang Zhang[1,4]*

Affiliations:

[1]Nanoscale Science and Engineering Center, University of California, Berkeley, CA, USA

[2]Department of Mechanical Engineering, Texas A&M University, College Station, TX, USA

[3]Department of Physics and Astronomy, University of California, Riverside, CA, USA

[4]Faculties of Sciences and Engineering, University of Hong Kong, Hong Kong SAR, China

*Correspondence to: Email: xzhang@me.berkeley.edu

This file includes:

Optical chirality in twisted bilayers

Asymmetric magneto-chiral behaviors

Supplementary Figs. 1-8

Optical chirality in twisted bilayers

Structural chirality is owing to the breaking of mirror symmetry, discerning an object to its mirror image, and hence those objects such as human hands are chiral. An intuitive chiral object is a helix because it breaks the mirror symmetry by twisting around while moving along an axis. For simplification yet keeping the physical picture, a common strategy is to artificially induce chirality by twisting two layers of structures, resembling only half of a pitch in a helix[1,2]. Like right hands fit to the right gloves, chiral objects preferably respond to a chiral stimulus with the same handiness. Same in the optical region that chiral materials respond preferably to left or right circularly polarized light depending on the handiness, because they are chiral stimuli[3,4]. In terms of absorption, a chiral response dictates that chiral materials absorb different amounts of the two circularly polarized light, which is also named circular dichroism.

When the constituting layers are atomically thin crystals, the twisted atomic bilayers also possess circular dichroism. Under the illumination of linearly polarized light that composes an equal amount of left and right circular polarization, the twisted atomic layers will absorb different amounts of the two circular components. The absorbed circularly polarized photons with high energy will further excite electrons in the valence band to the conduction band of the two valleys. Such a phenomenon is the conservation of the angular momentum of photons, governed by the valley selection rules in transition metal dichalcogenides (TMDs)[5,6]. As a result, the optical chirality manifests in valley polarization.

Asymmetric magneto-chiral behaviors

The optical chirality is so sensitive to the atomic registry that besides the twisting angle, the relative displacement or interlayer slip between atoms in the two layers also dramatically modifies the

chiral responses in terms of magnitude and spatial distribution[7,8]. Therefore, for a comprehensive knowledge of the optical chirality in twisted atomic bilayers, one has also to know the interlayer slip. In other words, two twisting bilayers with opposite twisting angles can be treated as partner structures but not necessarily mirror images because of the lack of information of the slip in them. Thus, solely by knowing the twisting angle, the two partner structures do not have to possess symmetric chiral behaviors, not even a fair comparison in terms of magnitude. Unfortunately, the twisting angle is relatively easy to control during fabrication but not the interlayer slip.

The exchange interactions of the magnetic proximity effect force the magneto-chiral response to deviate from the exact odd dependence on the magnetic field (**B**). In sharp contrast to bulk chiral materials, twisted atomic bilayers are vulnerable to the dielectric environment from a substrate. When placing the twisted atomic bilayers on a substrate, the wave functions in the top and bottom layers are no longer symmetric. Such an asymmetric spatial distribution in wave function along the wave propagation direction at the atomic scale is hardly observable under a uniform magnetic field for the magneto-chiral effect. However, it could be significant when using the magnetic proximity effect from a ferromagnetic substrate because the magnitude is proportional to the overlapping between the wave functions of the twisted layers and the magnetic field of the substrate. Therefore, when flipping the magnetic field in the substrate, the overlapping is different, resulting in the asymmetric behaviors of the magneto-chiral effect being not an exact odd function of the magnetic field. The asymmetric factor varies with the surface morphology of the ferromagnetic substrate.

Supplementary Fig. 1

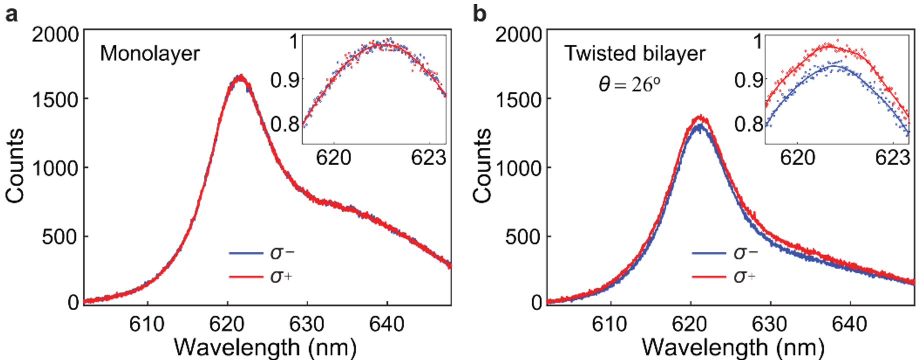

Supplementary Fig. 1 Twisting angle determines the chirality. a A monolayer WS_2 on a SiO_2/Si substrate has the degenerate states of the two valleys (σ^- and σ^+), which is due to the lack of geometrically induced chirality. This phenomenon verifies the experiment has corrected all system uncertainty that could lead to a difference between the two valleys. The inset is a close look at the center wavelength of the valleys with the intensity normalized to the local maximum. The solid line is a guide of the eye. **b** A twisted bilayer WS_2 with the twisting angle of 26° (clockwise) has a larger magnitude of σ^+ (red) than σ^- (blue), which reverses the chirality for that with a negative twisting angle of 28° (Figure 2c).

Supplementary Fig. 2

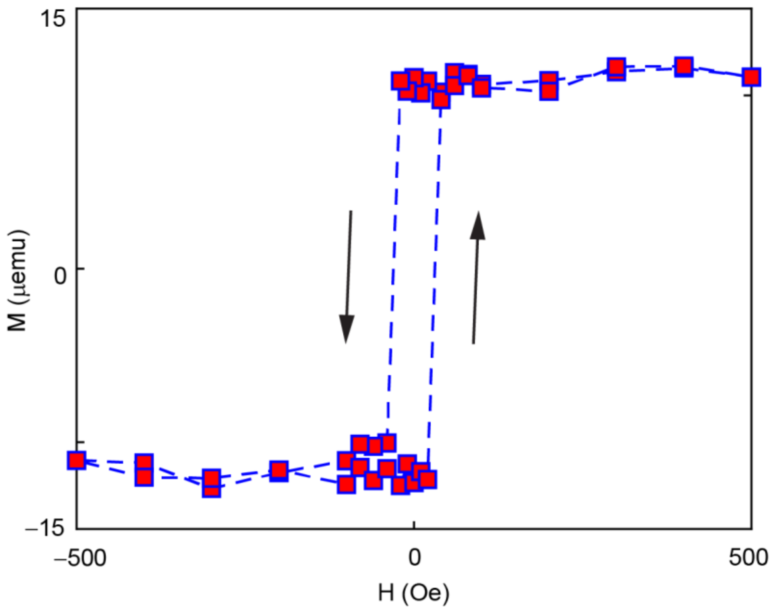

Supplementary Fig. 2 Ferromagnetic responses of TIG films at room temperature. The magnetic responses of the 10-nm thick TIG film grown on SGGG (111) by removing the linear paramagnetic background at room temperature unambiguously show a sharp hysteresis loop which is a signature of ferromagnetism. The out-of-plane magnetic moment provides a strong exchange magnetic field in the close vicinity of the surface. The narrow hysteresis loop indicates that the direction of the exchange magnetic field flips easily by applying a static magnetic field, such as placing a permanent magnet nearby.

Supplementary Fig. 3

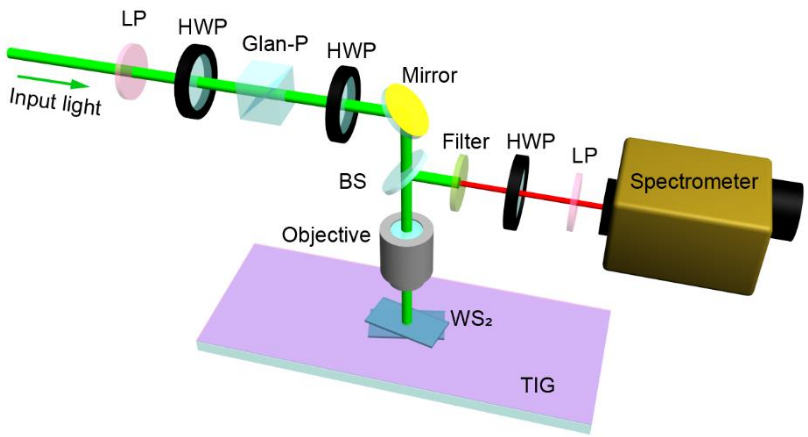

Supplementary Fig. 3 Schematic of the experimental setup for the photoluminescence and differential reflection spectroscopy. For the differential reflection measurements, we use a tungsten halogen white light source with a set of linear polarizers (LP) and half-wave plates (HWP) for tailoring the intensity and polarization of the input light. An HWP and LP in front of the spectrometer analyze the polariton of the light reflected from and sample and substrate. For the photoluminescence spectroscopy, ultrafast laser light pulses at the wavelength of 560 nm enter the system for the excitation of photoluminescence from the WS_2 atomic layers. A quarter-wave plate (not shown in the picture) between the beam-splitter (BS) and objective (40×, NA 0.65) not only circularly polarize the input light but also analyze the circular polarization components of the output photoluminescence.

Supplementary Fig. 4

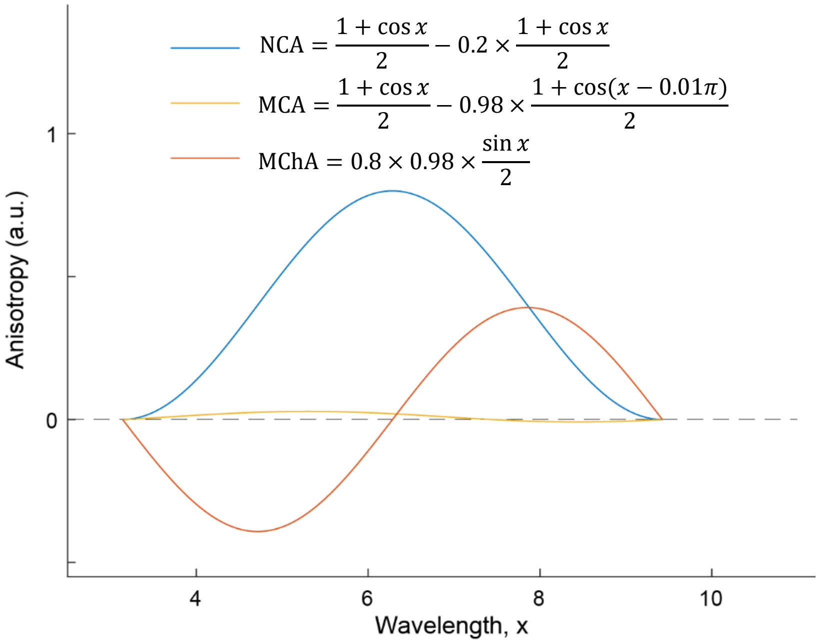

Supplementary Fig. 4 Oversimplified graphical illustration of the phenomenological model. For a graphical illustration, a shifted cosine function ($\frac{1+\cos x}{2}$) represents the photoluminescence spectrum. From the phenomenological model in the Methods section, the natural circular anisotropy (NCA) is a magnitude difference between the two photoluminescence spectra. As an example of illustration, the blue curve shows the exaggerated NCA by assuming the magnitude difference could reach a value of 0.8 while keeping the function unchanged. The magnetically induced circular anisotropy (MCA) slightly modifies both the magnitude (0.02) and phase (0.01π) of the photoluminescence spectra (yellow). Moreover, the magneto-chiral anisotropy (MChA) is a combination of the NCA and MCA while having the derivative dispersion with the sign determined by the relative direction between the magnetic moment and the wavevector (orange). Although both the phenomenological model and the graphical illustration are very simple, they help predict anisotropy behaviors such as the derivative dispersion relation in the experiment (Fig. 4c).

Supplementary Fig. 5

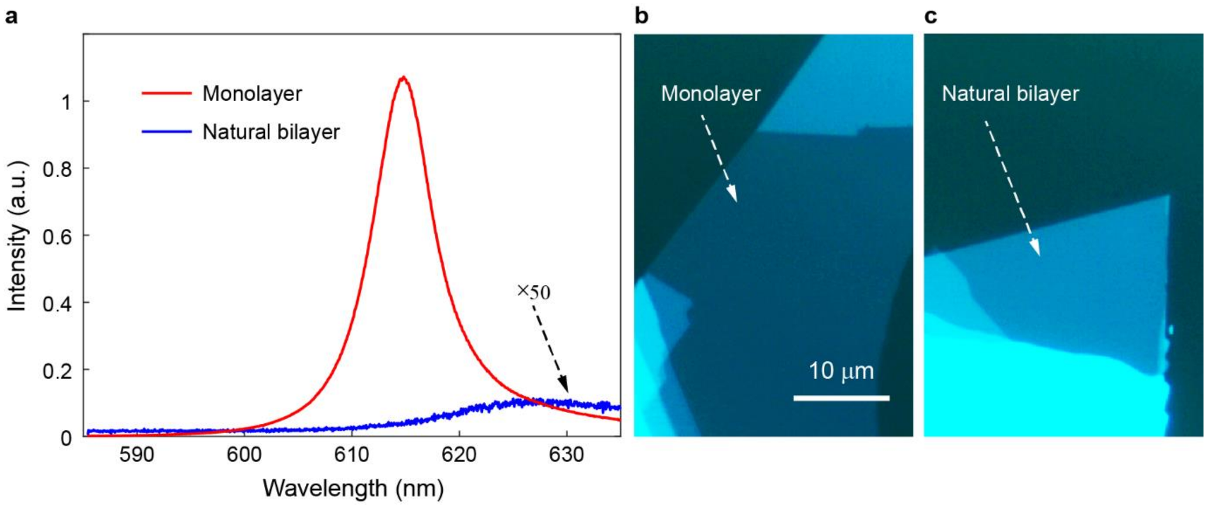

Supplementary Fig. 5 Photoluminescence spectra of tungsten disulfide (WS_2) atomic layers. **a** A significantly large peak at the wavelength of ~615 nm in the photoluminescence (PL) spectrum (red) shows the direct bandgap nature of the monolayer. The peak vanishes in the PL spectrum of the bilayer WS_2 (blue) signaling that it is an indirect bandgap semiconductor. The unique spectral feature at room temperature facilitates the identifying of WS_2 monolayers. **b** and **c** The optical image of the WS_2 atomic layers. The scale bar represents a length of 10 μm.

Supplementary Fig. 6

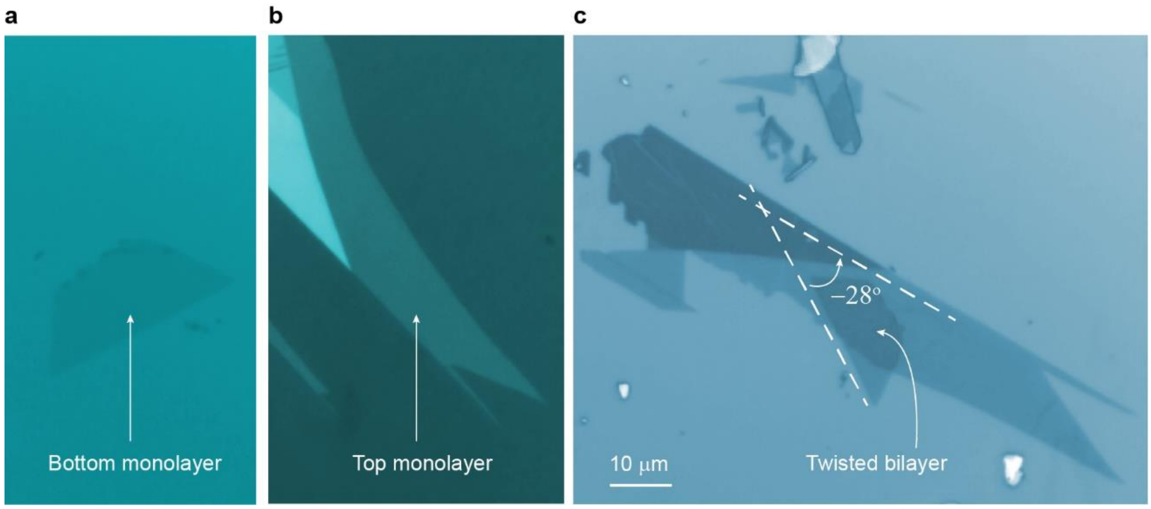

Supplementary Fig. 6 Optical images for a TBL-WS_2 with a negative twisting angle. a and **b** Optical images for the bottom and top ML-WS_2 before the twisting. We use PL spectra at room temperature to verify the monolayer feature of the samples. **c** The top ML-WS_2 twists counterclockwise (negative) referenced to the bottom ML-WS_2 with an angle of 28°. The structures with a negative and positive twisting angle represent the two counterparts (enantiomers) for chirality. The scale bar represents a length of 10 μm.

Supplementary Fig. 7

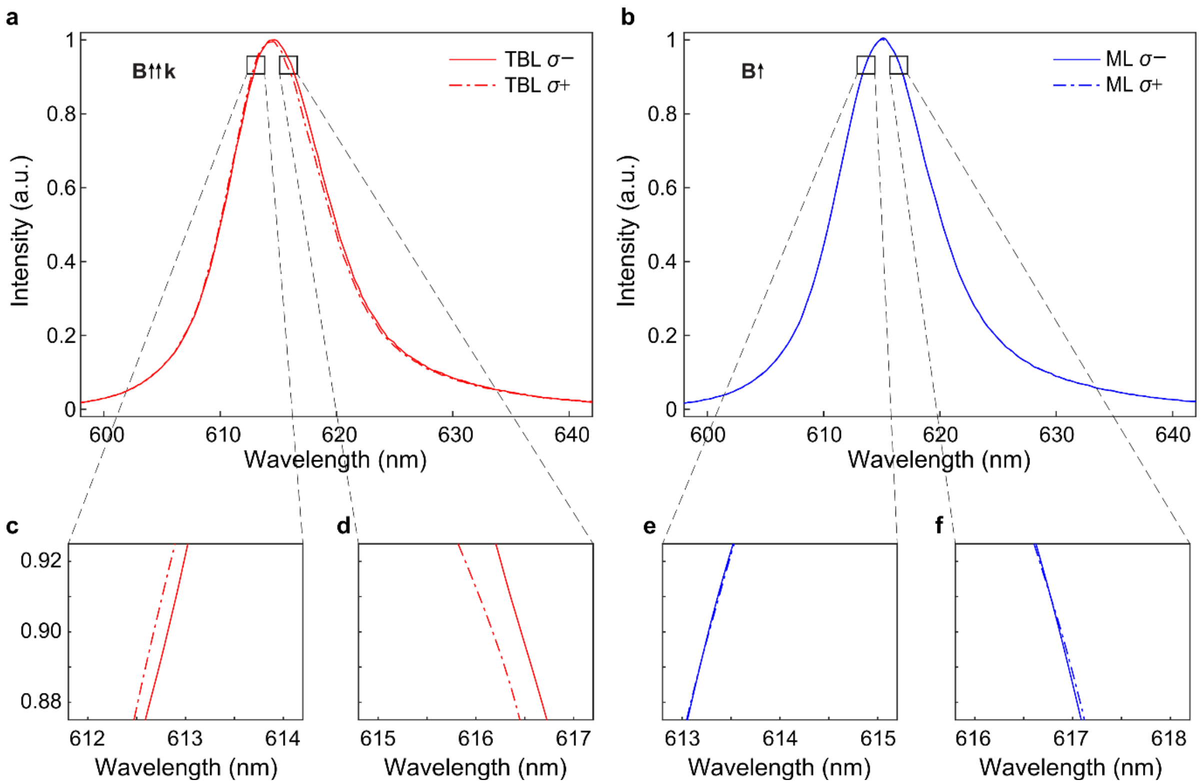

Supplementary Fig. 7 The raw spectra for acquiring the circular anisotropy plots. a The photoluminescence spectra for deriving the magneto-chiral anisotropy (MChA) with twisted bilayers on TIG when the magnetic moment and wavevector are in parallel (Fig. 4c, red). Compared to that in Fig. 2c for natural circular anisotropy (NCA), it is not straightforward to directly visualize the MChA from the raw spectra because of the MChA's derivative spectral behaviors. With a close look, **c** and **d** clearly show that the sign of the MChA flips across the peak. **b** The raw spectra for deriving the magnetically induced circular anisotropy (MCA in Fig. 4b, blue) with monolayer WS_2 on TIG can hardly see any differences. As expected, the indication of flipping sign disappears from the zoom-in spectra in **e** and **f**. The thermal broadening at room temperature and the dephasing effect of the valley excitons smear out the reduced spectral splitting (~0.2 nm). Nevertheless, by subtracting the two raw spectra in **a**, Fig. 4c (red) clearly shows the derivative dispersion in the magneto-chiral anisotropy spectrum, evidencing the observation of the magneto-chiral effect.

Supplementary Fig. 8

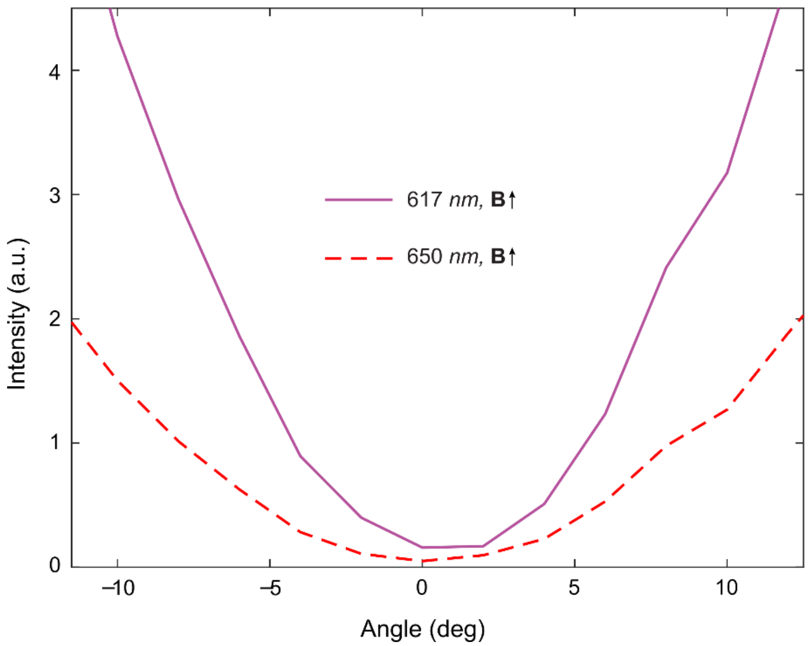

Supplementary Fig. 8 Magnetically induced polarization rotation on and off the exciton resonance. The differential reflection spectrum in Fig. 3d proves the excitonic effect, without which the peak should not exist. And hence, the polarization rotation under an exchange magnetic field at this peak using the same differential reflection spectrum must relate to the excitonic effect. As a control experiment, the dashed red curve analyzes the polarization rotation at a wavelength of 650 nm that is off-resonance. Compared to that at the peak wavelength (solid purple), the dashed red curve shows zero polarization rotation evident with a minimum at the angle of 0°. The polarization rotation of light with the magnetic substrate should not follow the dispersion of the excitonic resonance of WS_2, excluding the magnetic substrate being the cause.

References:

1 Zhang, S. *et al.* Negative refractive index in chiral metamaterials. *Phys. Rev. Lett.* **102**, 023901 (2009).

2 Cui, Y., Kang, L., Lan, S., Rodrigues, S. & Cai, W. Giant chiral optical response from a twisted-arc metamaterial. *Nano Lett.* **14**, 1021-1025 (2014).

3 Rogacheva, A. V., Fedotov, V. A., Schwanecke, A. S. & Zheludev, N. I. Giant gyrotropy due to electromagnetic-field coupling in a bilayered chiral structure. *Phys. Rev. Lett.* **97**, 177401 (2006).

4 Yin, X. H., Schaferling, M., Metzger, B. & Giessen, H. Interpreting chiral nanophotonic spectra: the plasmonic Born-Kuhn model. *Nano Lett.* **13**, 6238-6243 (2013).

5 Zeng, H. L., Dai, J. F., Yao, W., Xiao, D. & Cui, X. D. Valley polarization in MoS_2 monolayers by optical pumping. *Nat. Nanotechnol*. **7**, 490-493 (2012).

6 Mak, K. F., He, K. L., Shan, J. & Heinz, T. F. Control of valley polarization in monolayer MoS_2 by optical helicity. *Nat. Nanotechnol.* **7**, 494-498 (2012).

7 Addison, Z., Park, J. & Mele, E. J. Twist, slip, and circular dichroism in bilayer graphene. *Phys. Rev. B* **100**, 125418 (2019).

8 Chen, S. Z., Ling, X. H., Shu, W. X., Luo, H. L. & Wen, S. C. Precision measurement of the optical conductivity of atomically thin crystals via the photonic spin Hall effect. *Phys. Rev. Appl.* **13**, 014057 (2020).
